# Preparation and epitope mapping of broad-spectrum neutralizing monoclonal antibodies against economically important pestiviruses

**DOI:** 10.1186/s13567-026-01748-4

**Published:** 2026-05-15

**Authors:** Zhongdi Liu, Shijiang Mi, Sun He, Meng Wu, Fei Bao, Ye Feng, Zunbao Wang, Longfei Xu, Xiaomei Pan, Changchun Tu, Junhui Li, Wenjie Gong

**Affiliations:** 1 State Key Laboratory for Diagnosis and Treatment of Severe Zoonotic Infectious Diseases, Key Laboratory for Zoonosis Research of the Ministry of Education, College of Veterinary Medicine, Jilin University, Changchun, 130062 China; 2 State Key Laboratory of Pathogen and Biosecurity, Changchun Veterinary Research Institute, Chinese Academy of Agricultural Sciences, Changchun, 130122 China; 3 TECON Biopharmaceutical Co., Ltd., Urumqi, 830000 China; 4 Chongqing Academy of Animal Sciences, Chongqing, 400015 China; 5 Jiangsu Co-Innovation Center for Prevention and Control of Important Animal Infectious Diseases and Zoonoses, Yangzhou University, Yangzhou, 225009 China

**Keywords:** Pestivirus, neutralizing monoclonal antibodies, antigenic epitope, cross-neutralization

## Abstract

Pestiviruses are pathogens with broad host ranges, infecting domestic animals and wildlife, with some of them exhibiting antigenic similarity and cross-neutralization capability. However, the underlying molecular mechanism remains elusive. In this study, BALB/c mice were immunized with purified E2 protein of classical swine fever virus (CSFV) LPC strain to generate monoclonal antibodies (mAbs) using hybridoma technology. Immunofluorescence assay (IFA) and western blot were employed to determine the reactivity of mAbs with pestiviruses and E2 proteins. As a result, 31 mAbs against CSFV E2 protein were generated, with two mAbs TCH034 and TCH052 exhibiting broad-spectrum reactivity against different genotypes of CSFV, BVDV1 and BVDV2, as well as E2 proteins from 8 pestivirus species (CSFV, BVDV1, BVDV2, HobiPeV, BDV, AydinPeV, TSV and ovIT PeV). The pestivirus broad-spectrum mAbs demonstrated neutralizing activity against the available strains of CSFV, BVDV1, and BVDV2. Epitope mapping with truncated and point-mutated E2 proteins showed that TCH034 and TCH052 recognize a highly conserved antigenic epitope in E2 proteins among 8 pestivirus species, comprising residues G^113^, K^114^, W^133^ and G^135^, which was further confirmed by the inability of TCH034 and TCH052 to react with or neutralize the rescued CSFV strain JL23 containing substitutions K114M, K114N, K114R and K114T. Taken together, the findings of this study elucidate the molecular basis underlying antigenic conservation and cross-neutralization among pestiviruses, providing valuable support for the development of diagnostic assays targeting economically important pestiviruses.

**Supplementary Information:**

The online version contains supplementary material available at 10.1186/s13567-026-01748-4.

## Introduction

Pestiviruses exhibit a broad host range, capable of infecting not only domestic and wild even-toed ungulates, but also various wildlife species, such as gazelles, giraffes, pangolins, bats, and bamboo rats, as well as marine mammals like the harbor porpoise [1–5]. According to the latest taxonomic report published by the International Committee on Taxonomy of Viruses (ICTV) [6], the genus *Pestivirus* currently comprises up to 19 distinct viral species, including *Pestivirus bovis* (Bovine viral diarrhea virus 1, BVDV1), *Pestivirus tauri* (Bovine viral diarrhea virus 2, BVDV2), *Pestivirus suis* (CSFV), *Pestivirus ovis* (Border disease virus, BDV), *Pestivirus brazilense* (HoBi-like pestivirus, HoBiPeV or BVDV3), *Pestivirus aydinense* (AydinPeV), *Pestivirus N* (Tunisian sheep-like pestivirus, TSV), and *Pestivirus O* (ovine/IT pestivirus, ovIT PeV). Among these, CSFV, BVDV1, BVDV2, BVDV3 (HoBiPeV), and BDV, cause substantial economic losses in the livestock industry. Classical swine fever (CSF), caused by CSFV, is one of the most devastating swine diseases worldwide featuring high fever, hemorrhaging and reproductive disorders [7]. BVDV1 and BVDV2 infect a wide range of domestic and wild animals, including cattle, goats, sheep, pigs, deer, buffalo, bison, and alpacas, leading to diarrhea, respiratory and reproductive disorders, and persistent infections [8]. HoBiPeV, also referred to as BVDV3, first identified in contaminated bovine serum from Brazil [9], is prevalent in South America, Europe and Asia, causing respiratory distress, abortion, mucosal disease (MD), and gastrointestinal disorders [10]. BDV infection in sheep results in abortion, stillbirth, and immunosuppression [11]. In addition, BVDV1, BVDV2 and BVDV3, along with their corresponding neutralizing antibodies, are frequently present in bovine serum. Neutralizing antibodies against BVDV in bovine serum can reduce the infectious titers of pestiviruses with antigenic homology to BVDV, thereby interfering with the vaccine production of pestiviruses and related viral infection studies. Furthermore, the in vitro infection of susceptible cells with CSFV, BVDV, BDV and other pestiviruses is impaired when the culture medium is supplemented with bovine serum contaminated by pestiviruses. Collectively, bovine serum contaminated with pestiviruses and their neutralizing antibodies constitutes a major obstacle to life science research and pestivirus vaccine production, highlighting the necessity of stringent quarantine via pan-pestivirus detection methods.

For the initially defined pestiviruses, considerable genetic diversity was observed and each species of CSFV, BVDV, and BDV can be further classified into multiple genotypes [12–14]. Moreover, antigenic cross-reactivity is also present among different pestivirus species [15–17], but the molecular basis for this phenomenon remains poorly understood. So far, only a few broad-spectrum mAbs against pestiviruses have been reported, including m912 and 4-9D4 (targeting E2) [18, 19], 15C5 (E^rns^-specific) [20], and C16 (NS3-binding) [21]. The neutralizing mAb m912 could only react with BDV, GPeV and some CSFV strains, with recognition of key amino acid residues P/L^709^ and E^713^ [18]. The epitope ^995^YYEP^998^ recognized by mAb 4-9D4 exhibits high conservation in the E2 proteins of CSFV, BVDV1, BVDV2, BVDV3, and BDV, but efficient recognition of this epitope is only achieved after virion treatment with 1% NP-40 detergent, because the epitope is located at the protein C-terminus and close to the viral envelope, leading to insufficiently exposure to effectively stimulate humoral immunity and interact with antibody. Thereby the broad-spectrum application of mAb 4-9D4 in antigen and antibody detection was limited [19]. The E^rns^ protein mAb 15C5 exhibits reactivity towards the majority of BVDV1, BVDV2, BVDV3, BDV, and Bungowannah viruses [20]. Similarly, the NS3 protein mAb C16 demonstrates broad-spectrum recognition of pestiviruses [21]. However, the epitopes recognized by these non-neutralizing mAbs 15C5 and C16 have not been defined.

Here, we generated two broad-spectrum neutralizing mAbs (TCH034 and TCH052) targeting a conserved E2 epitope in strains of CSFV, BVDV1, BVDV2, BVDV3, BDV, and other 3 pestivirus species, providing novel insights into the structural basis for cross-neutralization among economically important pestiviruses. Our findings will also facilitate the development of both universal pestivirus antibody and antigen diagnostic tools.

## Materials and methods

### Cells and viruses

Porcine kidney cells (PK-15) maintained in our laboratory were cultured in minimum essential medium (MEM) supplemented with 8% fetal bovine serum (FBS). Madin-Darby bovine kidney (MDBK) cells were cultured in Dulbecco's modified eagle medium (DMEM) supplemented with 8% FBS. *Spodoptera frugiperda* cells (Sf9) were cultured in Grace’s Insect Medium supplemented with 4% FBS. PK-15 cells and MDBK cells were incubated at 37℃ in 5% CO_2_, while Sf9 cells were cultured at 27℃. Seventy-nine CSFV strains belonging to subgenotypes 1.1, 2.1, 2.2 and 2.3, four BVDV1 strains belonging to subgenotypes 1a, 1b, 1c, and 1 m, and one BVDV2a strain were stored in our laboratory.

### Preparation of hybridoma secreting E2 mAbs

The purified E2 protein of CSFV attenuated vaccine strain LPC provided by TECON Biopharmaceutical Co., Ltd was mixed with an equal volume of Seppic 201 adjuvant to achieve complete emulsification. Subsequently, female BALB/c mice aged 6–8 weeks were immunized via subcutaneous multi-site injections at 2-week intervals, with each mouse receiving 50 μg of protein per injection. One week after the third immunization, serum samples were collected. IFA was conducted to evaluate the reactivity of the diluted serum with LPC-infected cells, thereby determining the antibody titer in the sera of immunized mice. For the mice with the best immune response, 100 μg purified protein without adjuvant was injected intraperitoneally. One week later, spleen cells were collected and fused with myeloma cells SP2/0 by electrofusion method. After 10 days of culture in semisolid medium ClonaCell™-HY Cloning-Medium D (STEMCELL Technologies, Canada), independent cell clones were selected and cultured in 96-well plates for 72 h at 37 ℃ and 5% CO_2_. The hybridoma cells secreting mAbs against E2 protein were verified by IFA, and the positive cells were subcloned using semisolid medium. The hybridoma cells were verified by IFA and then expanded and cryopreserved. The animal experiments were approved by the Institutional Animal Care and Use Committee of Jilin University (no. KT202003064).

### Purification of mAbs from ascites

Female BALB/c mice, aged 6–8 weeks, were intraperitoneally injected with 300 μL of a dedicated adjuvant per mouse and reared for the next 12 days. Hybridoma cells were prepared by gently dispersion followed by centrifugation at 1000 × *g* for 5 min. After removing the culture supernatant, the cells were resuspended in normal saline solution to achieve a final cell density of 2 × 10^6^ cells/mL. Each mouse was then injected with 500 μL of resuspended hybridoma cells into the peritoneal cavity. Once the mice exhibited a significant increase in abdominal circumference, ascites was aspirated using a syringe, then centrifuged at 12 000 × *g* for 10 min. Finally, mAbs were purified from ascites using a Protein A/G 4FF chromatographic column followed by determination of antibody concentration.

### Identification of the reaction patterns between E2 mAbs and pestiviruses

A total of 79 CSFV strains representing different subgenotypes were collected from various regions of China between 1990 and 2019 and selected to characterize the reaction patterns between E2 mAbs and CSFV. These strains included 5 subgenotype 1.1 strains (encompassing the vaccine strains HCLV, LPC and the highly virulent Shimen strain), 42 subgenotype 2.1 strains, 22 subgenotype 2.2 strains, 10 subgenotype 2.3 strains. These viruses were inoculated into PK-15 cells at a multiplicity of infection (MOI) as 0.1. Separately, four BVDV-1 strains (1a, 1b, 1c, and 1 m) and one BVDV-2a strain were inoculated into MDBK cells at a MOI of 0.1 to characterize the reaction patterns between BVDV and E2 mAbs. Following incubation at 37℃ and 5% CO_2_ for 72 h, the infected cells were fixed with 80% cold acetone for 1 h. Subsequently, purified mAbs at a concentration of 1 mg/mL were diluted 1:1000 in PBS, and 100 μL of this dilution was added to each well as the primary antibodies. After incubation at 37 ℃ for 1 h and washing with PBS for 3 times, a secondary antibody of Alexa Fluor 488 donkey anti-mouse IgG (diluted to 1:500) was applied to the infected cells. The reactivity of CSFV and BVDV with the mAbs was visualized under a fluorescent microscope.

To further determine the reaction patterns between E2 mAbs and CSFV strains circulated in other countries and regions, E2 proteins derived from 18 CSFV representative strains covering 10 subgenotypes that circulate worldwide, which were expressed with the baculovirus expression system in our previous study [22], were used for analyzing the reactivity of mAbs with CSFV, with 20 μg for each protein applied. In addition, E2 proteins from representative strains of 17 different BVDV subgenotypes were also eukaryotically expressed as well as E2 proteins from representative strains of 9 other pestivirus species, excluding BVDV1-3 and CSFV (the strain information is presented in Additional file 1). The E2 proteins (1–331 aa) of BVDV and other pestivirus strains were synthesized and ligated into the pFastBac1 plasmid by the Comate Biotechology Company (Jilin, China), which were transformed into DH10Bac competent cells for the generation of recombinant baculoviral plasmids. Subsequently, the E2 proteins were expressed utilizing the insect baculovirus expression system, and 20–50 μg for each protein was then used for identifying the virus reaction spectrum of E2 mAbs through western blot as previously described [22]. The primary antibody, hybridoma cell culture supernatant, was diluted at a ratio of 1:100 and incubated with the E2 proteins transferred on the PVDF membrane overnight at 4 ℃, then incubated with the 1:500 diluted secondary antibody, Alexa Fluor 680 donkey anti-mouse IgG at room temperature for 1 h. The reaction patterns between mAbs and E2 proteins were determined through membrane scanning using Odyssey Imaging System.

### Neutralizing assay

Seven CSFV strains and five BVDV strains, each representing distinct subgenotypes and previously confirmed to react with mAbs TCH034 and TCH052, were diluted to a titer of 2000 TCID_50_/mL using MEM (for CSFV) or DMEM (for BVDV), respectively. Subsequently, 50 μL of each diluted viral suspension was aliquoted into individual wells in a 96-well plate. Purified mAbs TCH034 and TCH052 was adjusted to a concentration of 2 mg/mL, followed by twofold serial dilutions in MEM (for CSFV) or DMEM (for BVDV). Next, 50 μL of each antibody dilution was mixed with the corresponding viral solution and incubated at 37 °C for 1 h. Thereafter, 100 μL of PK-15 cells (for CSFV) or MDBK cells (for BVDV) were added to each well, and the plates were incubated at 37 ℃ with 5% CO_2_ for 3 days. IFA was then performed to evaluate the neutralizing activity of the mAbs against CSFV and BVDV. The neutralization titer of the mAbs against various CSFV and BVDV strains was defined as the highest dilution factor at which 50% of the replicate cell wells showed no viral infection (ND_50_). All generated ND₅₀ data were compiled and statistically analyzed using OriginPro 2025.

### Identification of antigenic epitopes recognized by TCH034 and TCH052

To identify the antigenic epitopes recognized by mAbs TCH034 and TCH052, LPC E2 protein (20 μg) was first treated with loading buffer supplemented with or without dithiothreitol (DTT), and western blot assays were subsequently performed to characterize the reactivity patterns of mAbs TCH034 and TCH052 against the differentially treated proteins, thereby distinguishing whether these two mAbs target linear or conformational epitopes. Subsequently, the epitope region targeted by TCH034 and TCH052 was localized through interaction between mAbs and the truncated variants of the CSFV E2 protein, which was eukaryotically expressed in our laboratory [22]. Western blot analysis was conducted with 20 μg for each truncated E2 protein to incubate with the mAbs for mapping the epitope-containing region. Following identification of the target region, E2 proteins derived from 50 representative pestivirus strains covering 11 species, including those exhibiting either reactive or non-reactive binding with the mAbs, were subjected to multiple sequence alignment using CLC Sequence Viewer 8.0, to identify candidate residues potentially responsible for the differential recognition. Subsequently, structural modeling of the E2 protein was performed using PyMOL to identify amino acid residues spatially adjacent to these candidate residues. After that, mutation of the screened residues in the E2 protein possibly responsible for binding to the mAbs was introduced through site-directed mutagenesis (SDM) using the Mut Express II Fast Mutagenesis Kit (Vazyme, China), with the plasmid pFastBac1-LPC-E2 [22] as the template and the primers detailed in Additional file 2 (LPC-G113H-F to LPC-W133A-R). The mutated E2 proteins were eukaryotically expressed with the insect baculovirus expression system as previously described [22]. Western blot analysis was then conducted to assess the binding capacity of mAbs TCH034 and TCH052 to these mutant E2 proteins, allowing the precise identification of the epitopes recognized by these two mAbs. To further validate the accuracy of the mapped epitopes, total RNA was extracted from hybridoma cells of TCH034 and TCH052 and served as the template for reverse transcription using the 5’ RACE method, the variable regions of the heavy and light chain genes were then amplified by PCR, the resulting amplicons were sequenced by Sangon Biotech (Shanghai) Co., Ltd.. The resulting antibody sequences were aligned with the CSFV E2 protein sequence using AlphaFold2 for epitope prediction.

To further verify the conservation of the antigenic epitope critical for the binding of these mAbs (TCH034 and TCH052) to the E2 protein, multiple sequence alignment was performed using E2 protein sequences derived from 1335 strains across 8 pestivirus species recognized by mAbs TCH034 and TCH052, including 265 strains of BVDV1, 162 strains of BVDV2, 828 strains of CSFV, 43 strains of BDV, 30 strains of HobiPeV, 2 strains of AydinPeV, 2 strains of TSV, and 3 strains of ovIT PeV. In addition, three pestivirus species that exhibited no reactivity with mAbs TCH034 and TCH052 (GPeV, PAPeV, and PPeV) were included as sequence references. For the differential sites, SDM was performed to mutate the corresponding amino acids in the E2 protein of CSFV subgenotype 2.1b strain JL23 based on the expression plasmid pcDNA3.1-JL23-E2 constructed in our laboratory and an N114K mutation was introduced into the GPeV E2 protein, the mutation primers (JL23-G113V-F to H138-N114K-R) are listed in Additional file 2. Subsequently, the mutant proteins were eukaryotically expressed as described above, the plasmids containing the site mutations were transfected into HEK-293 T cells using Lipofectamine 3000 (Thermo Fisher Scientific, USA). The expressed proteins were harvested at 72 h post-transfection and analyzed by western blot to determine whether the amino acid substitutions within the antigenic epitope affect the binding of the mAbs to the E2 protein.

### Rescue of epitope-mutated CSFV strain and evaluation of their reactivity with mAbs

Viral RNA was extracted from CSFV strain JL23 using the HiPure Viral RNA/DNA Kit (Magen, China), and cDNA was subsequently synthesized with the M-MLV reverse transcriptase (TaKaRa, China). The full-length viral genome was divided into six overlapping fragments (designated as S1 to S6), which were individually amplified by PCR using the specific primers (Additional file 3: JL23-F1-F to JL23-F6-R) and Vazyme P516 high-fidelity DNA polymerase (Vazyme, China). To construct JL23 mutant strains carrying the E2 substitutions K114M, K114N, K114R, or K114T, the wild-type fragment S3 was first cloned into the pFastBac1 vector, SDM was then performed using the Mut Express II Fast Mutagenesis Kit (Vazyme, China) to introduce the desired point mutations into the E2 genes. The mutagenesis primers are listed in Additional file 3 (JL23-K114M-F to JL23-K114T-R). Following sequencing validation of the mutated E2 genes via Sanger sequencing, the recombinant plasmids were used as templates for PCR amplification of the mutant S3 fragments containing the K114M, K114N, K114R, or K114T substitutions, respectively. To achieve viral genome circularization, a linker fragment is indispensable and was constructed with minor modifications based on previously reported protocols, with reference to the circular polymerase extension reaction (CPER) method developed by Tomokazu Tamura et al. for the rescue of BVDV [23]. This 1131-bp linker consists of the following sequential segments: a 20-bp sequence corresponding to the 3’-UTR terminus of the JL23 strain genome (AGGCAATTTCCTAACGGCCC), the hepatitis D virus ribozyme, the SV40 polyadenylation (SV40 polyA) signal, a 165-bp spacer, the cytomegalovirus (CMV) promoter, and a 36-bp sequence mapping to the 5’-UTR terminus of the JL23 strain genome (GTATACGAGATTAGCTCATCCTCGTGTACAATATTG). The linker fragment was artificially synthesized and inserted into the plasmid pFastBac1 at the restriction sites between *Bam*H I and *Eco*R I. The resulting recombinant plasmid was subsequently utilized as the template for the linker fragment amplification. Following this, the amplified linker fragment was assembled with six overlapping genomic fragments of the virus to generate circular DNA, with the assembly reaction performed using Vazyme P516 high-fidelity DNA polymerase.

Co-cultured PK-15 and HEK-293 T cells were transfected by circular DNA using Lipofectamine LTX (Invitrogen, USA), followed by incubation at 37 °C with 5% CO_2_ for 3 days prior to passage. During passage, HEK-293 T cells were selectively eliminated owing to their distinct trypsin sensitivity relative to PK-15 cells, thereby enabling exclusive propagation of CSFV in PK-15 cells. To assess the stable propagation potential of the recombinant CSFV strains in PK-15 cells, serial passages were conducted. Subsequently, the full-length E2 gene of the virus at Passage 6 was amplified by RT-PCR with the primer sets listed in Additional file 3 (outer primers: CSFV-E2-WF and CSFV-E2-WR; inner primers: CSFV-E2-NF and CSFV-E2-NR), and the amplified products were subjected to Sanger sequencing. Following confirmation of stable viral replication, growth curves of the recombinant viruses were constructed to evaluate the effect of the K^114^ mutations in the E2 protein on viral replication kinetics. PK-15 cells were inoculated separately with the mutant and wild-type viruses at a MOI of 0.1, and cell culture supernatants were harvested at 12, 24, 48, and 72 hours post-infection (hpi) for viral titration, which was performed in triplicate for each sample. Mean values and standard deviations were calculated based on the data from three independent experiments, and graphs were generated using OriginPro 2025. To determine the influence of the K^114^ mutations on the binding reactivity between mAbs and the recombinant viruses, the interactions between mAbs TCH034/TCH052 and the rescued mutant or wild-type CSFV strains were analyzed by IFA. For the assessment of mAb neutralizing activity against the mutant CSFV strains, 10 μg of purified TCH034 and TCH052 derived from ascites were incubated with 100 TCID_50_ of the parental or recombinant CSFV JL23 strains. Hybridoma cell SP2/0 ascites and CSFV E2-neutralizing mAb HCL-001 ascites served as the as negative and positive controls, respectively [24].

### Phylogenetic analysis of pestiviruses

To characterize the phylogenetic relationship of pestivirus strains that exhibit either reactivity or non-reactivity with mAbs TCH034 and TCH052, E2 protein gene genes from 30 representative strains covering all 19 pestivirus species were retrieved from GenBank. The selected strains comprised 4 BVDV1 strains, 3 BVDV2 strains, 5 CSFV strains, 2 BDV strains, and single strains of 15 additional pestivirus species: PAPeV, PPeV, GPeV, HoBiPeV, AydinPeV, RPeV, APPeV, LindaV, PhoPeV, TSV, ovIT PeV, DYPV, RtNn-PeV, RtAp-PeV, and BtSk-PeV. Phylogenetic analysis was conducted using the maximum-likelihood (ML) method implemented in MEGA 7.0 software [25, 26], and the robustness of the inferred phylogenetic tree was evaluated via 1000 bootstrap replicates to ensure statistical reliability. Subsequent post-processing and visual optimization of the phylogenetic tree were performed using the tvBOT for clearer presentation of evolutionary relationship [27].

## Results

### TCH034 and TCH052 exhibit broad-spectrum reactivity with pestiviruses

**Figure 1:**
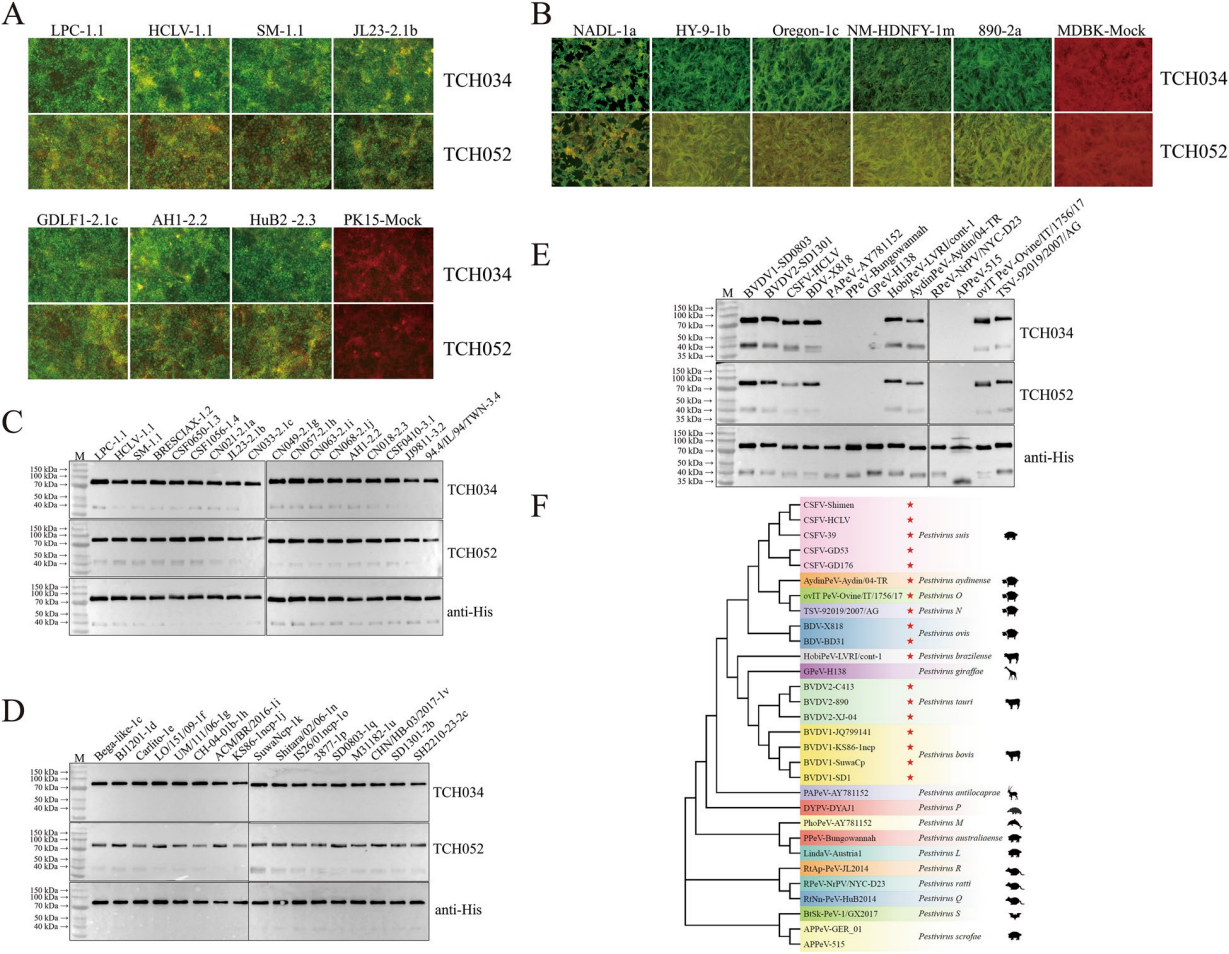
**Reaction patterns of mAbs TCH034 and TCH052 with pestiviruses**. **A** reactivity of TCH034 and TCH052 with CSFV. IFA was performed with 79 CSFV strains covering subgenotypes 1.1, 2.1, 2.2, 2.3 to assess the reactivity of the mAbs with CSFV, and the mAbs showed positive binding to all tested CSFV strains, with the reactivity profiles against 7 representative CSFV strains presented. **B** cross-reactivity of TCH034 and TCH052 with BVDV1 strains NADL-1a, HY-9-1b, Oregon-1c, NM-HDNFY-1 m and BVDV-2a strain 890. IFA results showed that both TCH034 and TCH052 exhibited positive reactivity with all tested BVDV strains. **C** reactivity of TCH034 and TCH052 with CSFV E2 proteins derived from 18 strains covering 10 distinct subgenotypes. Western blot with 20 μg for each protein demonstrated that TCH034 and TCH052 displayed robust binding to all tested E2 proteins. **D** cross-reactivity of mAbs TCH034 and TCH052 with 17 BVDV E2 proteins with each representing a unique subgenotype. Western blot was conducted with 20 μg for each protein, except those of strains BJ1201-1d, UM/111/06-1 g, and SD1301-2b (30 μg). The results confirmed the broad-spectrum reactivity of the mAbs with BVDV strains. **E** reactivity of TCH034 and TCH052 with 13 pestivirus E2 proteins with each representing a unique species. Western blot with 20 μg for each protein (30 μg for BDV‑X818 and APPeV‑515 and 50 μg for PAPeV‑AY781152, PPeV‑Bungowannah, and RPeV‑NrPV/NYC‑D23) indicated that the two mAbs cross-reacted with the E2 proteins of 8 different pestivirus species. F, phylogenetic tree of pestiviruses based on full-length E2 gene sequences of 30 pestivirus strains representing 19 species. Phylogenetic analysis was conducted using MEGA 7.0 with the Maximum-likelihood method and JTT matrix-based model [25, 26], and the phylogenetic tree was visualized and optimized using tvBOT [27]. Pestivirus strains recognized by TCH034 and TCH052 were labeled with red asterisks.

Using hybridoma technology, a total of 31 hybridoma clones secreting antibodies specifically targeting the CSFV LPC strain were isolated from 2,700 hybridoma cell clones via IFA using LPC-infected PK-15 cells. Subsequently, IFA was performed to verify the reactivity of the resulting mAbs against 79 CSFV strains spanning four subgenotypes (1.1, 2.1, 2.2, and 2.3). Among these mAbs, TCH034 and TCH052 reacted with all tested CSFV strains, and their reactivity against representative strains of the four subgenotypes was presented in Figure 1A. Unexpectedly, TCH034 and TCH052 also cross-reacted with BVDV strains available in our laboratory, including BVDV-1a (NADL), BVDV-1b (HY-9), BVDV-1c (Oregon), BVDV-1 m (NM-HDNFY) and BVDV2a (890) (Figure 1B), demonstrating their broad reactivity toward multiple pestivirus species. Moreover, the reactivity intensity of TCH034 against all CSFV and BVDV strains was significantly stronger than that of TCH052 (Figures 1A and B).

To further confirm the broad-spectrum reactivity of TCH034 and TCH052 against CSFV, the E2 proteins of CSFV representative strains belonging to six subgenotypes (1.2, 1.3, 1.4, 3.1, 3.2, and 3.4) that circulated in other countries and regions were eukaryotically expressed, along with those of CSFV strains prevalent in mainland China (subgenotypes 1.1, 2.1, 2.2, and 2.3), the latter included most CSFV strains used in the aforementioned IFA (Figure 1A). Western blot analysis revealed that both TCH034 and TCH052 bound to the E2 proteins of all 10 CSFV subgenotypes with comparable affinity. Prominent bands corresponding to the E2 dimer were observed, and faint bands of the E2 monomer were also detected (Figure 1C). Furthermore, western blot was conducted to determine the reactivity of TCH034 and TCH052 against the E2 proteins of BVDV1 (15 subgenotypes) and BVDV2 (2 subgenotypes), excluding the 5 BVDV strains used in the IFA (Figure 1B). As shown in Figure 1D, E2 dimeric bands were detected for all tested BVDV E2 proteins following incubation with TCH034 and TCH052. Notably, TCH052 exhibited weaker binding activity than TCH034 toward BVDV E2 proteins. The above results suggested that TCH034 and TCH052 may possess pan-pestivirus reactivity, which was further validated by western blot using E2 proteins of representative strains from 13 pestivirus species. As depicted in Figure 1E, in addition to BVDV1, BVDV2, and CSFV, both mAbs also bound to the E2 proteins of pestiviruses infecting domestic animals (swine, cattle, and sheep), including BDV (*Pestivirus ovis*), AydinPeV (*Pestivirus aydinense*), HobiPeV (*Pestivirus brazilense*, BVDV3), TSV (*Pestivirus N*), and ovIT PeV (*Pestivirus O*) (Figure 1F). In contrast, no reactivity was detected against pestivirus strains primarily circulating in wildlife, including PAPeV (*Pestivirus antilocaprae*), PPeV (*Pestivirus australiaense*), GPeV (*Pestivirus giraffae*), RPeV (*Pestivirus ratti*), or APPeV (*Pestivirus scrofae*)-the only swine-associated pestivirus among this wildlife-associated group (Figure 1F). Additionally, the pestiviruses recognized by these two mAbs are of great economic importance to the livestock industry, whereas those not detected exhibited extremely limited epidemiological distribution worldwide.

### TCH034 and TCH052 could neutralize CSFV, BVDV1, and BVDV2

**Figure 2:**
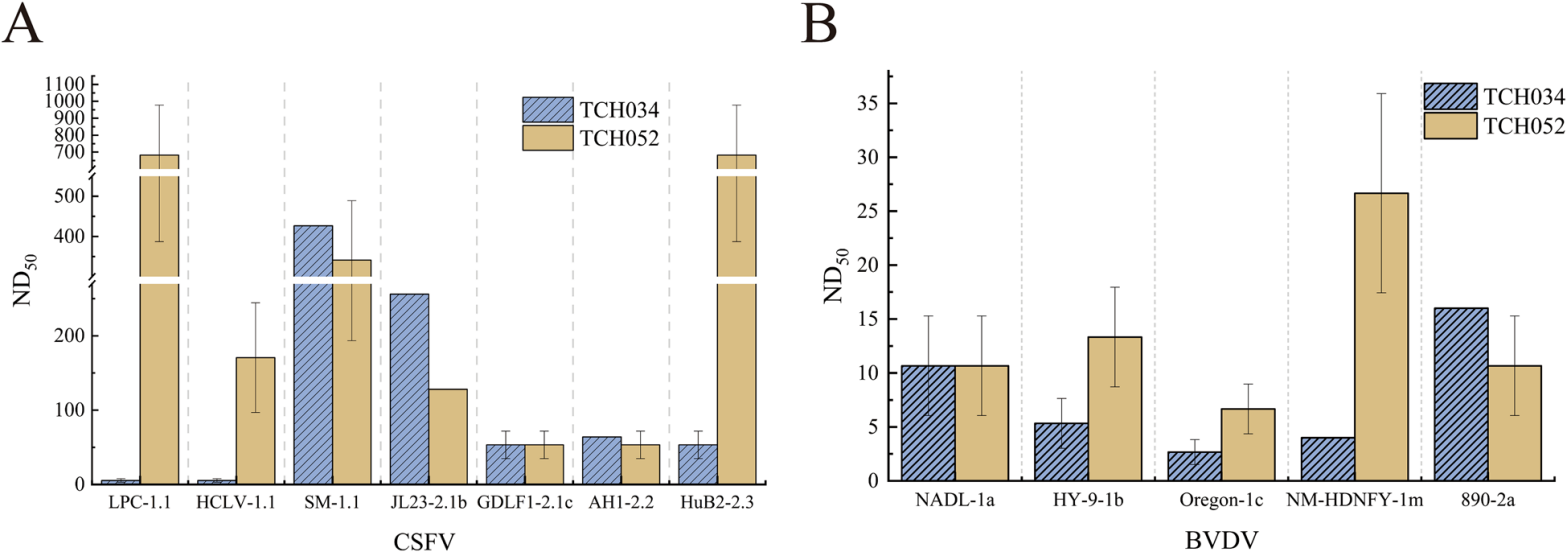
**Determination of the neutralizing efficacy of mAbs TCH034 and TCH052 against diverse CSFV (A) and BVDV (B) strains**. Briefly, 2 μg of purified mAbs were serially two-fold diluted, 100 μL of each dilution was then incubated with 100 TCID_50_ of CSFV or BVDV strains at 37 ℃ for 1 h. The mixture was subsequently inoculated onto PK-15 cells (for CSFV) or MDBK cells (for BVDV) cultured in 96-well plate. After incubation for 72 h, IFA was conducted to determine the ND_50_ of the mAbs against each viral strain. The results revealed that TCH034 and TCH052 exerted potent neutralizing activity against both CSFV and BVDV isolates. Bar heights denote the mean ND₅₀ values, and error bars represent the standard deviation (SD) calculated from three independent experimental replicates.

To assess the neutralizing activity of the broad-spectrum pestivirus mAbs TCH034 and TCH052, the two mAbs were first purified from ascites using a protein A/G affinity chromatography, with their concentration subsequently quantified. Neutralization assays were then performed using purified mAbs TCH034 and TCH052 to evaluate their neutralizing potency against the CSFV and BVDV strains detailed in Figures 1A and B. Briefly, 2 μg of each mAbs was two-fold serially diluted and incubated with 100 TCID_50_ of the respective diluted viruses. As shown in Figure 2A, both TCH034 and TCH052 neutralized CSFV, yet with markedly distinct efficiencies. Notably, TCH034 showed weaker neutralization against the CSFV attenuated vaccine strains HCLV/LPC (ND_50_ = 4–8) relative to other CSFV strains (ND_50_ = 32–512). In contrast, the ND_50_ of TCH052 for neutralizing LPC and HCLV was 512–1024 and 128–256 respectively. As depicted in Figure 2B, both mAbs also neutralized BVDV, while the maximum ND_50_ of the two mAbs against different BVDV strains ranging from 16 to 32. A comparative analysis of their neutralizing capacity against CSFV and BVDV revealed that TCH034 and TCH052 exhibited significantly stronger neutralization against CSFV than BVDV, with the only exception being the weak neutralization of the CSFV vaccine strains HCLV and LPC by TCH034. Interestingly, although TCH034 showed higher reactivity with all tested CSFV and BVDV strains than TCH052 in IFA (Figures 1A and B), TCH052 demonstrated superior neutralizing activity against specific CSFV and BVDV strains.

### Conserved epitope recognized by TCH034 and TCH052

**Figure 3:**
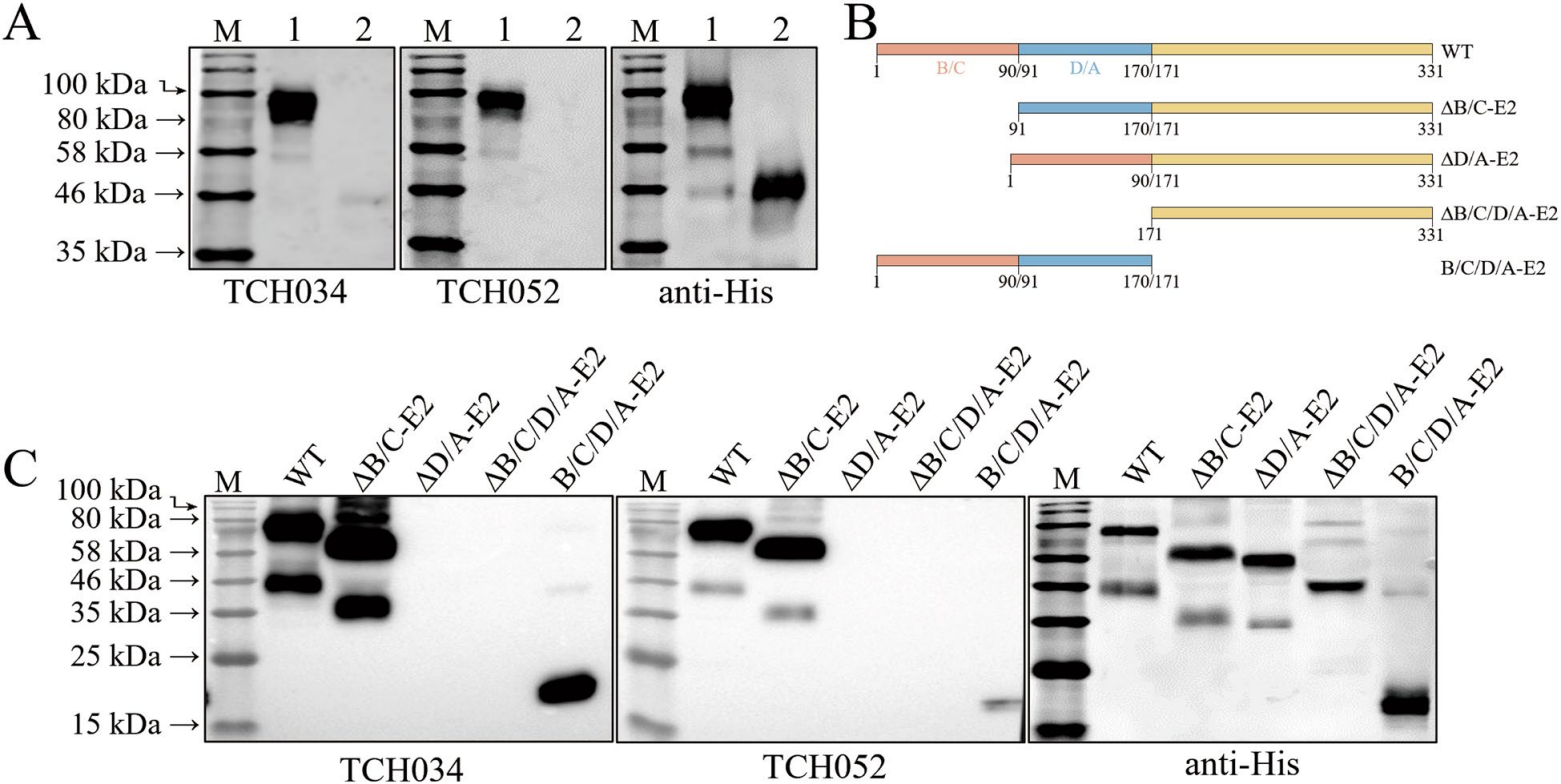
**Identification of antigenic regions recognized by mAbs TCH034 and TCH052**. **A** reactivity of mAbs TCH034 and TCH052 with the E2 protein under different buffer conditions: Lane 1, untreated E2 protein without DTT incubation; Lane 2, reduced E2 protein following DTT treatment. **B** Schematic representation of wild-type and truncated E2 proteins: WT, full-length E2 protein; ∆B/C-E2, E2 protein with B/C antigenic domain deletion; ∆D/A-E2, E2 protein with D/A antigenic domain deletion; ∆B/C/D/A-E2, E2 protein with B/C/D/A antigenic domain deletion; B/C/D/A-E2, B/C/D/A antigenic domain of E2 protein. **C** binding reactivity of TCH034 and TCH052 with truncated E2 proteins. Wild-type and four truncated E2 variants were eukaryotically expressed, and western blot analysis was performed using the two mAbs, with 20 μg of total protein loaded per lane. The results clearly demonstrated that the binding epitopes of TCH034 and TCH052 map to the D/A antigenic domain of the E2 protein.

To determine whether the broad-spectrum mAbs recognized linear or conformational epitopes, the CSFV-LPC E2 protein was treated with loading buffer, either with or without reducing reagent DTT, followed by western blot analysis. As shown in Figure 3A, the reaction between the mAbs and the E2 protein was abolished upon DTT treatment, confirming that the mAbs specifically recognized conformational epitopes. Next, truncated CSFV E2 proteins lacking the main antigen regions [22], the schematic diagram of which are illustrated in Figure 3B, were reacted separately with TCH034 and TCH052. Western blot revealed that neither TCH034 nor TCH052 bound to the truncated E2 protein lacking the D/A antigenic region, reflecting that both mAbs recognize an epitope located within the D/A region (Figure 3C).

**Figure 4:**
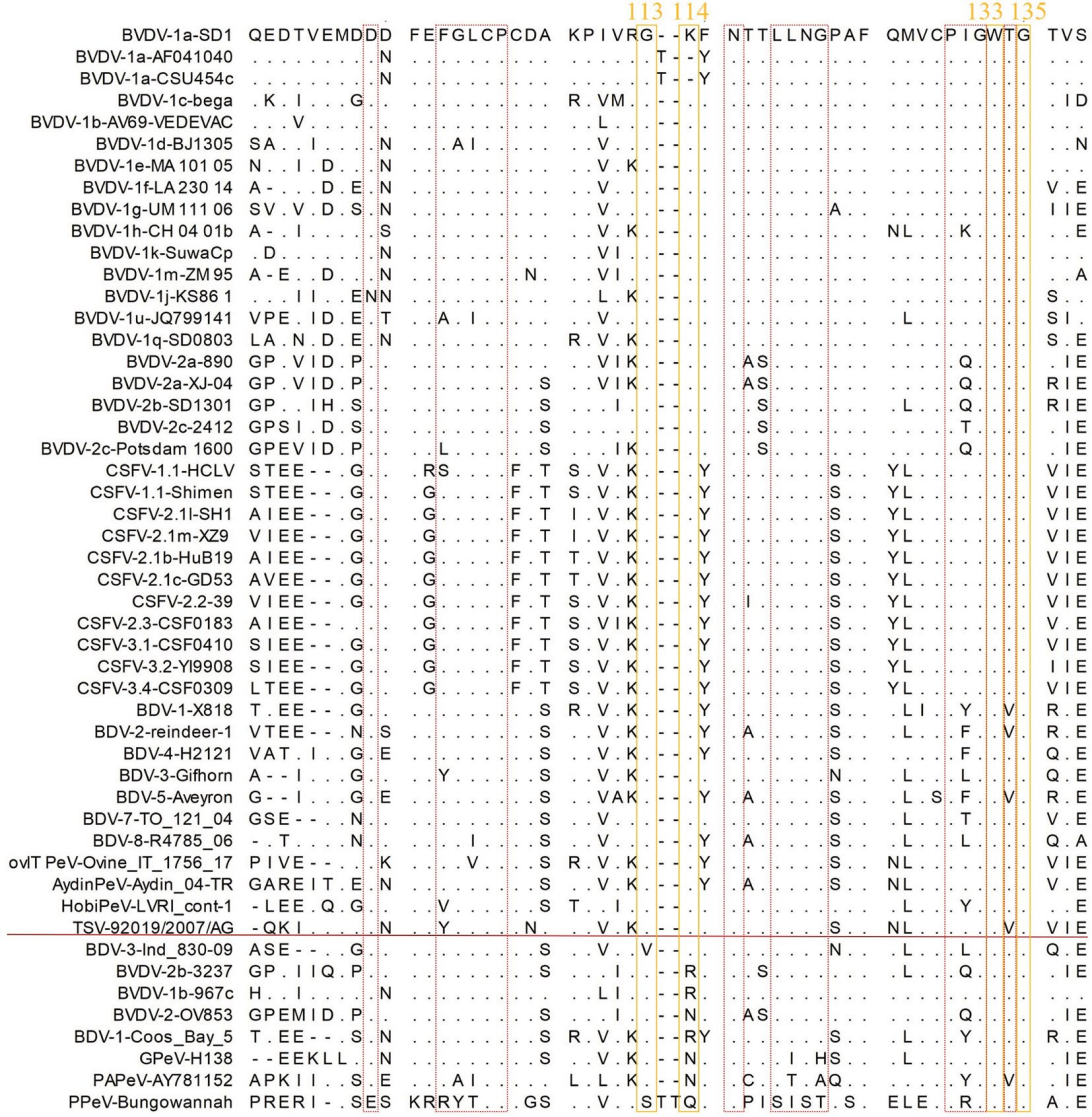
**Alignment of deduced amino acid sequences of pestivirus E2 proteins**. Multiple sequence alignment was performed for E2 proteins of 50 pestivirus strains derived from 11 species using CLC Sequence Viewer 8.0, with the E2 protein of BVDV-1a-SD1 serving as the reference sequence. Residues identical to those in the reference sequence are denoted by dots. Pestivirus E2 proteins positioned above the solid red line are recognized by mAbs TCH034 and TCH052, whereas those located below the solid red line exhibit no binding reactivity with these two mAbs. Residues that impair mAb-protein binding upon mutation are highlighted in yellow boxes, while residues non-essential for mAb binding are marked in red boxes.

**Figure 5:**
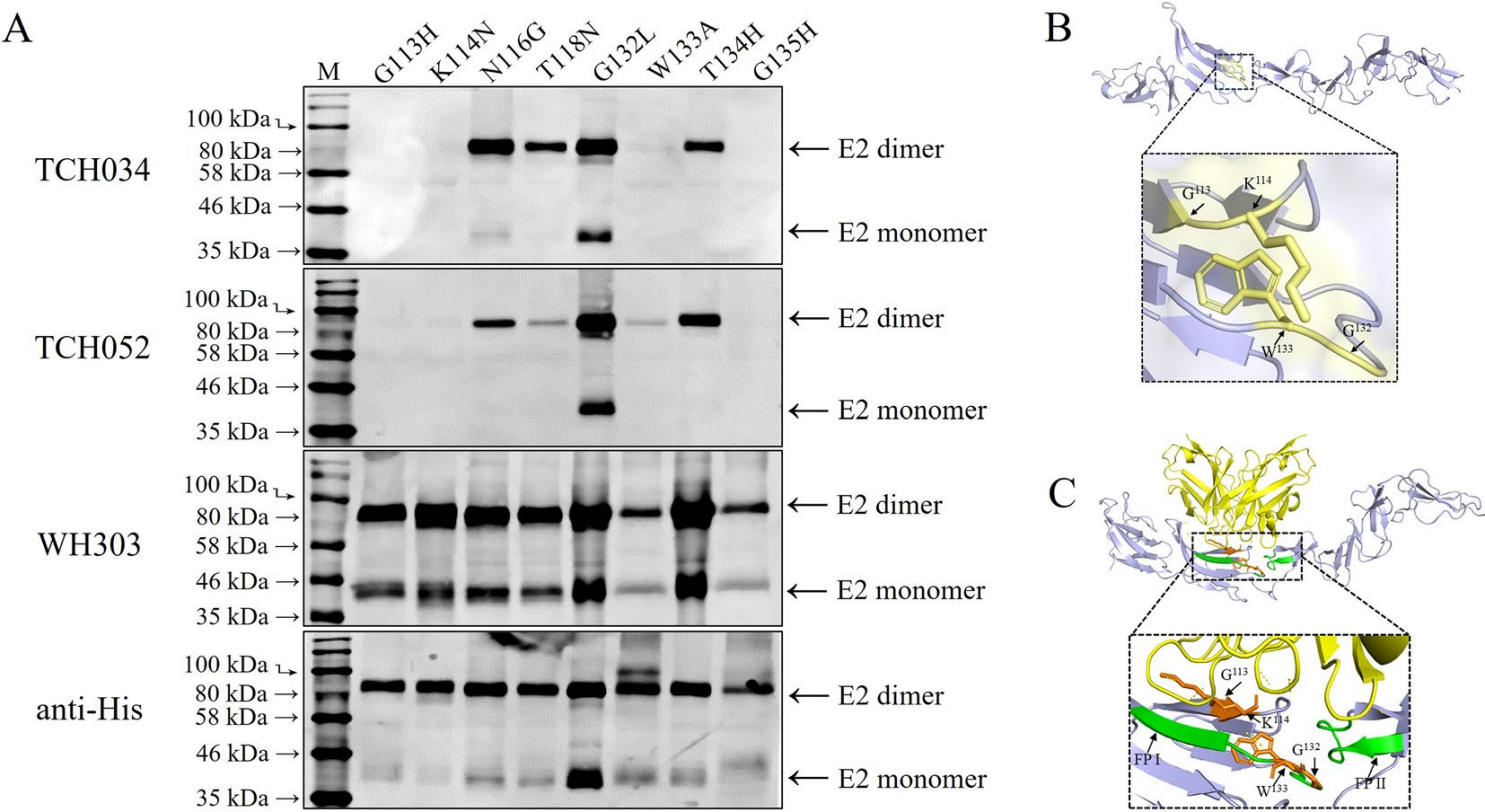
**Antigenic epitope recognized by TCH034 and TCH052**. **A** identification of amino acid residues targeted by TCH034 and TCH052. LPC-E2 proteins harboring site-directed mutations (G113H, K114N, N116G, T118N, G132L, W133A, T134H, and G135H were eukaryotically expressed using the insect baculovirus expression system. Twenty micrograms of each mutated protein harvested from the culture supernatants was subjected to SDS-PAGE and western blot to assess the binding reactivity with TCH034 and TCH052, with E2-specific mAb WH303 and anti-His mAb employed as controls. The results revealed that the G113H, K114N, W133A, and G135H substitutions abolished the binding of TCH034 and TCH052 to the E2 proteins, demonstrating that residues G^113^, K^114^, W^133^, and G^135^ form the core antigenic epitope recognized by these two mAbs. **B** structural simulation of the antigenic epitope (G^113^, K^114^, W^133^, and G^135^) targeted by mAbs TCH034 and TCH052, generated using PyMOL software. **C** AlphaFold-predicted binding interface between mAb TCH034 and the CSFV E2 protein. Color coding: yellow (mAb), orange (epitope), and green (fusion peptide segments).

Sequence alignment of the D/A regions of E2 proteins across different strains demonstrated that strains reacted with TCH034 and TCH052 harbor a lysine residue (K) at position 114 in the E2 protein, whereas the pestivirus strains that are not detected by the mAbs carry asparagine (N) or glutamine (Q) at this site (Figure 4). Structural modeling of the E2 protein further indicated that amino acid residues 113, 116, 118, 132, 133, 134, and 135 were spatially adjacent to K^114^. To further investigate the binding site for the broad-spectrum mAbs, a series of LPC-E2 proteins with site-directed mutations (G113H, K114N, N116G, T118N, G132L, W133A, T134H, and G135H) were expressed using the insect baculovirus system. Western blot showed that mutations at G113H, K114N, W133A, and G135H abrogated reactivity of the E2 proteins with both TCH034 and TCH052 (Figure 5A). As a control, WH303 [28], another mAb targeting the D/A region of CSFV E2 protein, also exhibited reduced binding to W133A and G135H mutants, suggesting that these two residues probably influence the overall antigenic structure of the D/A region. Notably, mutations at other conserved sites within this region did not affect antibody binding. Therefore the epitope recognized by TCH034 and TCH052 comprises residues G^113^K^114^, W^133^and G^135^ in the E2 protein, with G^113^ and K^114^ critical for direct antigen–antibody binding, and W^133^ and G^135^ likely maintain the structural integrity necessary for epitope presentation, which was further verified by both PyMOL-based epitope modeling (Figure 5B) and structural simulation between TCH034 and the E2 protein using AlphaFold2 (Figure 5C), in the latter K^114^ resides within the antibody-binding interface, forming hydrogen bonds with the mAb. The residues W^133^ and G^135^ affecting the binding of the broad-spectrum pestivirus mAbs to the E2 proteins were previously identified to be located within the fusion peptide 1 (FP1), which plays a crucial role in the fusion process between the viral envelope and the cellular membrane [29, 30].

To further verify the conservation of the critical antigenic epitope responsible for the binding of TCH034 and TCH052 to the E2 protein, multiple sequence alignment was performed with the E2 proteins derived from 1,335 pestivirus strains spanning 8 distinct species (CSFV, BVDV1, BVDV2, HobiPeV, BDV, AydinPeV, TSV, and ovIT PeV), all of which show reactivity with the two mAbs. The results revealed that the residues within the antigenic epitope recognized by TCH034 and TCH052 were highly conserved (98.6%, 1316/1335) across these mAb-reactive pestivirus species, with the exception of amino acid substitutions at positions 113 and 114 in 19 strains (1.4%, 19/1335), and multiple sequence alignment of a subset of representative strains is shown in Figure 4. Specifically, at site 113, one BDV strain exhibited a G113V substitution. At site 114, the K114R substitution was observed in 7 strains (3 BDV, 3 BVDV1, and 1 BVDV2), K114T in 5 BVDV1 strains, K114M in 3 BVDV1 strains, and K114N in 3 BVDV2 strains. Notably, these amino acid substitutions (G113V, K114R, K114T, K114M, and K114N) were found to abolish the binding interaction between the CSFV JL23 E2 protein and mAbs TCH034/TCH052 (Additional file 4). Furthermore, we observed that the GPeV strain H138 E2 protein harboring the N114K substitution gained reactivity with these mAbs (Additional file 4), further validating that residue K^114^ is critical for the binding of these mAbs to pestivirus E2 proteins.

### Recovery and characterization of recombinant epitope-mutated CSFV

**Figure 6:**
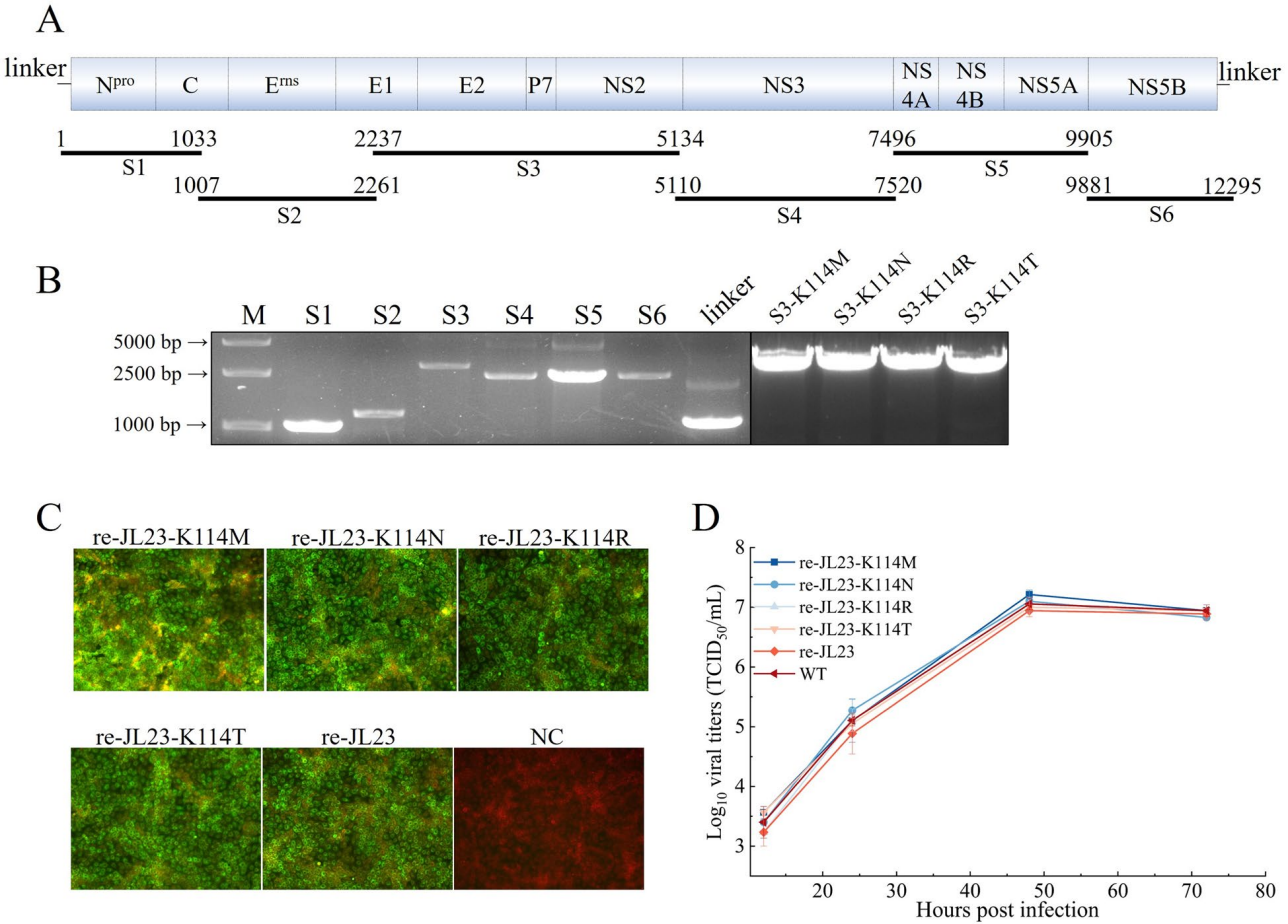
**Recovery and characterization of recombinant epitope-mutated viruses**. **A** strategy for the construction of a full-length cDNA clone derived from the CSFV strain JL23. The viral genome was divided into six overlapping fragments (designated S1-S6) to facilitate molecular cloning. **B** agarose gel electrophoresis analysis of PCR amplicons corresponding to fragments S1-S6, the synthetic linker, and individual mutated S3 fragments harboring K114M, K114N, K114R, K114T substitutions. **C** identification of recombinant parental and mutant JL23 viruses. All recombinant JL23 strains were rescued via CPER as previously described [23]. PK-15 cells were inoculated with the 6th passage of each recombinant viral stock, and viral infection was subsequently detected by IFA. For IFA staining, mAb WH303 served as the primary antibody, while Donkey anti-Mouse IgG (H + L) Highly Cross-Adsorbed Secondary Antibody, Alexa Fluor™ 488 was used as the secondary antibody. The results confirmed the successful rescue of both the recombinant parental virus and all mutant viruses. **D** growth kinetics of wild-type and mutant CSFV JL23 strains. PK-15 cells were infected with each recombinant JL23 strain at a MOI of 0.1. The viral cultures were harvested at 12, 24, 48, and 72 hpi for viral titration. Notably, all mutant JL23 strains displayed replication kinetics comparable to that of the parental strain, with no significant differences in viral replication capacity observed.

To further verify the epitope targeted by the mAbs and explore its effects on viral replication, the wild-type CSFV strain JL23 (subgenotype 2.1b) and a panel of mutant viruses harboring single amino acid substitutions (K114M, K114N, K114R, and K114T) within the E2 protein were rescued via CPER [23]. The full-length genome of the JL23 strain was amplified as six overlapping fragments following the strategy depicted in Figure 6A. Following amplification of these genomic fragments (Figure 6B), Fragment S3 was subcloned into the pFastBac1 vector, which was then utilized as the PCR template to introduce the individual mutations K114M, K114N, K114R, and K114T into the E2 genes. After validation via Sanger sequencing, mutant version of Fragments S3 carrying the targeted mutations were generated by PCR using the sequence-confirmed mutated plasmids as templates. The viral genomic fragments and linker were cyclized via CPER, and the resulting circularized products were subsequently transfected into PK-15 and HEK-293 T cells. The recombinant virus-infected cells were serially passaged five times, and successful virus rescue was confirmed by IFA. As depicted in Figure 6C, robust fluorescent signals were detected in cells infected with both the wild-type and mutant JL23 strains, verifying the successful rescue of recombinant viruses designated re-JL23 (wild-type), re-JL23-K114M, re-JL23-K114N, re-JL23-K114R, and re-JL23-K114T, respectively. Sanger sequencing of the E2 gene from the 6th passage viral cultures revealed that all recombinant mutant viruses carried no extraneous mutations, with only the designed amino acid substitutions at site 114 of the E2 protein detected. This result confirmed the genetic stability of these recombinant viruses during serial passage in PK-15 cells. To evaluate the impact of the epitope mutations on viral replication, PK-15 cells were inoculated with each recombinant virus at a MOI of 0.1. As illustrated in Figure 6D, the results demonstrated that mutation of residue K^114^ within the antigenic epitope recognized by mAbs TCH034 and TCH052 exerted no significant effect on viral replication. Notably, the viral propagation capacity of all mutant strains was comparable to that of the parental wild-type JL23 strain.

### Epitope mutation abolished the reaction between mAbs and the epitope-mutated CSFV strain

**Figure 7:**
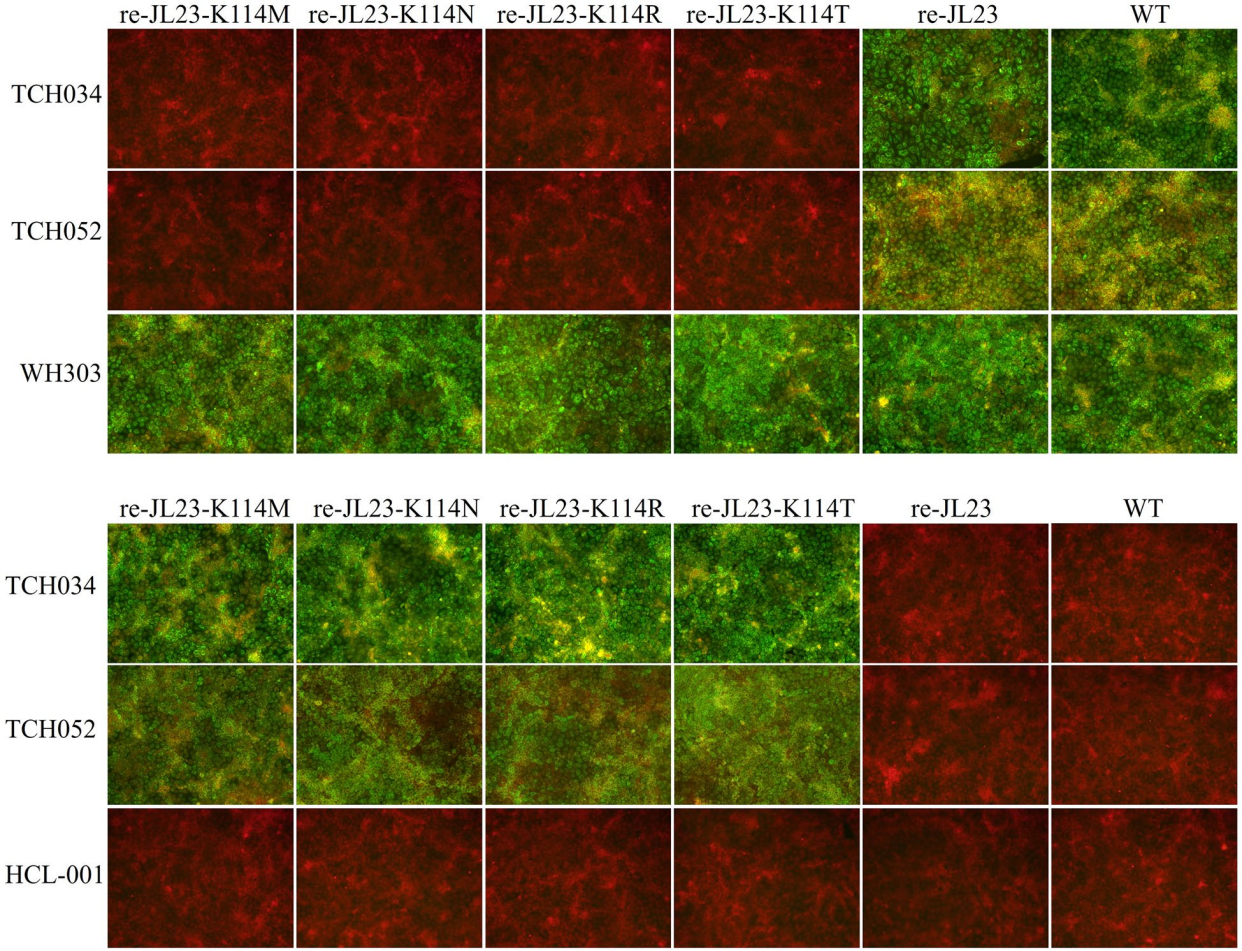
**Epitope mutation abrogated the binding of mAbs TCH034 and TCH052 and the recombinant mutant viruses**. **A** mutations of residue K^114^ abrogated the reactivity of TCH034 and TCH052 with the parental and mutant JL23 strains. PK-15 cells were inoculated with the parental or mutant JL23 strains and incubated for 72 h, after which viral infection was detected by IFA using mAbs TCH034 and TCH052 as primary antibodies, with WH303 serving as the positive control. IFA results revealed that K114M, K114N, K114R, K114T substitutions abrogated the reactivity of the two mAbs to the replicating viruses. **B** mutation of residue K^114^ abrogated the neutralizing activity of TCH034 and TCH052 against the recombinant mutant viruses. Purified TCH034 or TCH052 (10 μg for each) was incubated with 100 TCID_50_ of the parental or mutant JL23 strains for 1 h, with HCL-001 as the positive control. The mixtures were then inoculated into PK-15 cells. After incubation for 72 h, viral neutralization was evaluated by IFA. The results demonstrated that the recombinant JL23 strains carrying K114M, K114N, K114R, K114T mutations in the E2 protein could not be neutralized by mAbs TCH034 and TCH052.

IFA was performed to evaluate the reactivity of TCH034 and TCH052 with these recombinant mutant viruses, with the parental strain JL23 and mAb WH303 serving as controls. As shown in Figure 7A, PK-15 cells infected by either the parental or recombinant viruses exhibited green fluorescence following incubation with mAb WH303, confirming the successful infection of PK-15 cells by all viral strains. As expected, mAbs TCH034 and TCH052 can react with the parental JL23 strain and its non-mutant recombinant derivatives; in contrast, no discernible green fluorescence was detected in cells infected with any of the mutant viruses after incubation with these two mAbs, These findings indicated that mutation of the K^114^ residue in the E2 protein abrogated the reactivity of TCH034 and TCH052 with the replicative viruses. Neutralization assays were further conducted to assess the ability of TCH034 and TCH052 to neutralize the parental and recombinant viruses, with the broad-spectrum neutralizing mAb HCL-001 (targeting the CSFV E2 protein) used as a positive control [24]. As shown in Figure 7B, strong green fluorescence signals were sustained in PK-15 cells infected with the K^114^-mutated virus following treatment with TCH034 and TCH052. Collectively, these results demonstrate that the K^114^ mutations abrogated the neutralizing activity of the two mAbs against the mutant viruses, thereby further confirming that the K^114^ residue is a critical component of the antigenic epitope recognized by TCH034 and TCH052.

## Discussion

Up to date 19 species have been identified within the *pestivirus* genus, some of them infecting pigs, cattle, sheep and goats are widely distributed and cause substantial economic losses, such as CSFV, BVDV1, BVDV2, BVDV3, and BDV [7–11], while the others are mainly derived from wildlife and less prevalent, thus little damage to the domestic livestock [1–5]. Sequence comparison indicated that CSFV together with the pestiviruses infecting cattle, sheep and goat are closely related to each other and clustered together in the phylogenetic tree compared to those infecting wildlife (Figure 1F), for instance, CSFV strains share 56.5%-72.8%/54.2%-76.1% nucleotide and amino acid sequence homology of E2 genes with BVDV1, BVDV2, BVDV3, BDV, TSV and ovIT PeV, but far distant from others with 37.4%-59.7%/22.7%-57.4% nucleotide and amino acid sequence homology respectively, reflecting that genetic and antigenic similarity in the viral dominant antigen E2 protein are present between CSFV and pestiviruses infecting cattle and sheep, and cross-neutralization reactivity was also observed among these pestiviruses. However, the molecular basis for cross-neutralization reactivity among livestock-damaging pestiviruses has not yet been fully elucidated. To date, only two broad-spectrum neutralizing mAbs targeting the E2 proteins of pestiviruses have been reported, recognizing the epitopes P/L^709^ and E^713^ [18, 31]. However, these two mAbs specifically recognized BDV, GPeV, and certain strains of CSFV, but showed no reactivity with BVDV, a widespread and highly pathogenic virus in even-toed ungulates. In addition, although mAb 4-9D4 recognizes multiple members of the *Pestivirus* genus capable of infecting even-toed ungulate animals, no neutralizing ability was reported probably because of the location of 4-9D4-recognized epitope ^995^YYEP^998^ in the C-terminus of E2 protein and close to the viral envelope [19]. In this study, two pestivirus broad-spectrum mAbs TCH034 and TCH052 against E2 protein were generated and found to be reacted with 8 pestivirus species infecting pigs and cloven-hoofed ruminant animals, which also exhibit neutralizing capacity against various strains of CSFV, BVDV1 and BVDV2. While cell-adapted strains of BVDV3 and ovine pestiviruses were unavailable in our laboratory, or indeed anywhere in China, such strains could theoretically be requested from laboratories outside the country. However, the quarantine requirements for importing viral pathogens from foreign countries are extremely stringent, making it impossible to perform neutralization assays with these ruminant pestiviruses. Nevertheless, the antigenic epitope recognized by TCH034 and TCH052 is highly conserved across all CSFV and other mAb-reacted pestiviruses. Furthermore, comparative analysis of E2 proteins revealed that CSFV shares a closer evolutionary relationship with BVDV3 and four ovine pestivirus species-including BDV, AydinPeV, TSV, and ovIT PeV [10, 14–17], with amino acid sequence homology ranging from 61.4% to 76.1%, which is notably higher than that observed between CSFV and BVDV1-2 (53.9%-61.7%). Based on these findings, we propose that these mAbs may also be capable of neutralizing other pestiviruses that infect even-toed ungulates, beyond BVDV1 and BVDV2. Therefore, the findings obtained in this study firstly deciphered the molecular basis for antigenic cross reactivity or even cross-neutralization among CSFV and economically important ruminant pestiviruses, which will benefit for elucidating the cross immune reaction mechanism of pestivirus vaccines.

Novel ruminant pestiviruses are continually being identified [32], thus developing pan-ruminant pestivirus diagnostic assays are important for surveillance, prevention and control of pestivirus-associated diseases. In addition, the quality of commercial bovine serum was significantly impaired by the presence of various BVDVs and their antibody, the latter are commonly considered “contaminant” in the serum [33], leading to the severe damage of development of life science research and vaccine production, because the multiplication of CSFV and BVDV in the cells can be influenced by both the pestiviruses and their neutralizing antibodies. In this case, quarantine of the bovine serum products is essential with the pan-pestivirus detection kits, but none is commercially available, especially those capable of detecting the neutralizing antibodies against pestiviruses. Using the pan-pestivirus neutralizing mAbs TCH034 and TCH052, a blocking ELISA and a double antibody sandwich ELISA can be developed for the detection of pestivirus antibodies and antigens, respectively. These assays are applicable for the quarantine of bovine serum and epidemiological surveillance of pestiviruses. If pestivirus antibodies are detected in bovine serum, subsequent accurate diagnosis of the specific pestivirus can be implemented using antibody detection kits or methods specific to BVDV1, BVDV2, BVDV3 and even BDV. The pan-pestivirus antigen detection kit enables the screening of both important and emerging pestiviruses closely related to those recognized by TCH034 and TCH052. Follow-up testing with pestivirus-specific detection methods or kits can then be performed to identify the exact viral species, obviating the need for separate testing of each sample. This strategy markedly reduces both the time and economic costs associated with pestivirus detection. Furthermore, the conservative neutralizing antigenic epitope recognized by the broad-spectrum mAbs is considered potential target for the development of pan-pestivirus vaccine. Thus, the obtained pestivirus broad-spectrum mAbs in this study exhibit great application potential.

Based on the IFA results, the fluorescent intensity of TCH034 binding to pestiviruses was higher than that of TCH052. However, the neutralizing efficacy of TCH034 and TCH052 against pestiviruses (CSFV and BVDV) showed no correlation with their respective fluorescent intensities. In certain cases, TCH052 exhibited more potent neutralizing activity, demonstrating that the binding affinity of these mAbs is not consistent with their neutralization capacity. This discrepancy has also been reported previously on antibodies targeting the Spike protein of SARS-CoV-2 [34]. Furthermore, despite the high conservation of the antigenic epitope among pestiviruses reactive to these mAbs, both TCH034 and TCH052 displayed variable neutralization capacity against different CSFV and BVDV strains. The distinct binding affinity and neutralizing potency of the two mAbs are likely attributable to the significant differences in their aa sequences. Sequence alignment analysis revealed that TCH034 and TCH052 had amino acid variations at 8/11, 1/3, and 5/10 positions in the complementary-determining regions 1, 2 and 3 (CDR1, CDR2, CDR3) of the light chain, respectively, and at 5/8, 2/8, and 9/15 positions in the CDR1, CDR2, and CDR3 of the heavy chain, respectively. Notably, we observed an unexplained phenomenon wherein TCH034 showed extremely low neutralizing efficacy against the E2 protein-derived CSFV attenuated vaccine strain LPC, yet it could effectively neutralize other CSFV field isolates-including the SM strain, which is closely related to LPC. In contrast, TCH052 was able to neutralize all tested CSFV strains, including the vaccine strains LPC and HCLV and all field isolates analyzed. To address these aforementioned questions, the biological characteristics of TCH034 and TCH052, as well as the molecular mechanism underlying the interaction between these mAbs and pestiviruses, should be comprehensively investigated in future studies.

It was reported that CSFV E2 protein encompasses two fusion peptides (FPs) exhibiting membrane fusion activity, which are essential for the viral replication, and these two FPs are located at ^129^CPIGWTGVIEC^139^ and ^180^CKWGGNWTCV^189^ in the E2 protein [29, 30], respectively. Through docking by AlphaFold2, we found that the two FPs could not be fully displayed after antibody binding to E2 protein, which may disrupt virus-cell membrane fusion, this mechanism likely underlies the neutralizing activity of the two broad-spectrum pestivirus mAbs, which should be validated by elucidating the structure of mAb-E2 protein complex. Additionally, the observation of viral neutralization following mAbs binding to the residues within the FPs provides orthogonal validation of the critical role of FPs in mediating viral entry into host cells.

## Supplementary Information

**Additional file 1. Information of pestivirus strains with E2 protein expression.**

**Additional file 2. Primers used for the preparation of the SDM E2 protein.**

**Additional file 3. Primers used for the construction and validation of the recombinant CSFV strains.**

**Additional file 4. Reactivity of the site-mutant E2 proteins with mAbs TCH034 and TCH052.** The CSFV-JL23 E2 protein, which naturally reacts with mAbs TCH034 and TCH052, was subjected to SDM. Specifically, residue G113 in the JL23 strain E2 protein was mutated to V^113^, while residue K^114^ was mutated to T^114^, R^114^, M^114^, and N^114^, respectively. The E2 proteins with amino acid substitutions at 113 and 114 were transiently expressed in HEK293T cells as well as the E2 protein (N114K) of GPeV strain H138, and 20 μg of total protein in the culture supernatants for each protein was subjected to SDS-PAGE and western blot. As a result, the epitope-localized mutations abrogated the binding of the JL23 E2 proteins to both mAbs. In parallel, the N114K mutation in the E2 protein of GPeV strain H138 conferring binding reactivity to both mAbs.

## Data Availability

The corresponding data are all presented in this article, and the remaining materials or reagents are available from the authors upon reasonable request.
