# Supplementary material for: Preparation and epitope mapping of broad-spectrum neutralizing monoclonal antibodies against economically important pestiviruses
Source: Vet Res. 2026 May 15;57:74. doi: 10.1186/s13567-026-01748-4 (PMC13179623; doi:10.1186/s13567-026-01748-4)
Supplement: Supplementary file 1 — Additional file 1. Information of pestivirus strains with E2 protein expression. [file 13567_2026_1748_MOESM1_ESM.pdf]

Additional file 1. Information of pestivirus strains with E2 protein expression

Species	Virus	Isolate	genotype	GenBank accession number
Pestivirus bovis	BVDV	XH32	1a	
Pestivirus bovis	BVDV	Bega-like	1c	KF896608
Pestivirus bovis	BVDV	BJ1201	1d	KT943518
Pestivirus bovis	BVDV	Carlito	1e	KP313732
Pestivirus bovis	BVDV	LO/151/09	1f	MW054939
Pestivirus bovis	BVDV	UM/111/06	1g	MW054936
Pestivirus bovis	BVDV	CH-04-01b	1h	MW655625
Pestivirus bovis	BVDV	ACM/BR/2016	1i	KX857724
Pestivirus bovis	BVDV	KS86-1ncp	1j	AB078950
Pestivirus bovis	BVDV	SuwaNcp	1k	KC853440
Pestivirus bovis	BVDV	Shitara/02/06	1n	LC089876
Pestivirus bovis	BVDV	IS26/01ncp	1o	LC089875
Pestivirus bovis	BVDV	3877	1p	MW013505
Pestivirus bovis	BVDV	SD0803	1q	JN400273
Pestivirus bovis	BVDV	M31182	1u	JQ799141
Pestivirus bovis	BVDV	CHN/HB-03/2017	1v	ON901785
Pestivirus tauri	BVDV	890	2a	NC_039237
Pestivirus tauri	BVDV	SD1301	2b	KJ000672
Pestivirus tauri	BVDV	SH2210-23	2c	HG426494
Pestivirus suis	CSFV	LPC	1.1	
Pestivirus suis	CSFV	HCLV	1.1	Z46258
Pestivirus suis	CSFV	SM	1.1	AY775178
Pestivirus suis	CSFV	BRESCIAX	1.2	AY578687
Pestivirus suis	CSFV	CSF0650	1.3	JX028200.1
Pestivirus suis	CSFV	CSF1056	1.4	JX028202.1
Pestivirus suis	CSFV	CN021	2.1a	
Pestivirus suis	CSFV	CN069	2.1b	
Pestivirus suis	CSFV	CN033	2.1c	
Pestivirus suis	CSFV	CN049	2.1g	
Pestivirus suis	CSFV	CN057	2.1h	
Pestivirus suis	CSFV	CN063	2.1i	
Pestivirus suis	CSFV	CN068	2.1j	
Pestivirus suis	CSFV	CN002	2.2	
Pestivirus suis	CSFV	CN018	2.3	
Pestivirus suis	CSFV	CSF0410	3.1	JQ411575
Pestivirus suis	CSFV	JJ9811	3.2	KF669877

Pestivirus suis	CSFV	94.4/IL/94/TWN	3.4	AY646427
Pestivirus ovis	BDV	X818	1	AF037405
Pestivirus antilocaprae	PAPeV			AY781152
Pestivirus australiaense	PPeV	Bungowannah		NC 023176
Pestivirus giraffae	GPeV	H138		AF144617
Pestivirus brazilense	HobiPeV	Th/04_KhonKaen		NC_012812
Pestivirus aydinense	AydinPeV	04-TR		NC 018713
Pestivirus ratti	RPeV	NrPV/NYC-D23		NC 025677
Pestivirus scrofae	APPeV	515		NC 038964
Pestivirus N	TSV	92019/2007/AG		MZ664274
Pestivirus O	ovIT PeV	Ovine/IT/1756/17		MG770617.1
