# Supplementary material for: Preparation and epitope mapping of broad-spectrum neutralizing monoclonal antibodies against economically important pestiviruses
Source: Vet Res. 2026 May 15;57:74. doi: 10.1186/s13567-026-01748-4 (PMC13179623; doi:10.1186/s13567-026-01748-4)
Supplement: Supplementary file 2 — Additional file 2. Primers used for the preparation of the SDM E2 protein. [file 13567_2026_1748_MOESM2_ESM.pdf]

Additional file 2. Primers used for the construction of the SDM protein

Primer	Sequence (5'-3')
LPC-G113H-F	CCGTTGTCAAGCATAAGTACAATGCGACCTTGGTGAA
LPC-G113H-R	CTTATGCTTGACAACGGGTCTCGTATCAAACG
LPC-K114G-F	CAAGGGAGGTTACAATGCGACCTTGGTGAACG
LPC-K114G-R	CATTGTAACCTCCCTTGACAACGGGTCTCGTA
LPC-W133A-F	ATAGGGGCGACGGGTGTTATAGAGTGCACAGCAGTGAGCC
LPC-W133A-R	AACACCCGTCGCCCCTATTGGGCAGACAAGATAGAAAGC
LPC-G135H-F	GTGGACGCATGTTATAGAGTGCACAGCAGTGAGCC
LPC-W133A-R	CTATAACATGCGTCCACCCTATTGGGCAGACA
JL23-G113V-F	GGTGAAGGTGAAGTACAACACCACCCTGCTGAA
JL23-G113V-R	TGTACTTCACCTTCACCACGGGGGTTGTGTCG
JL23-K114T-F	GAAGGGGACATACAACACCACCCTGCTGAACG
JL23-K114T-R	TGTTGTATGTCCCCTTCACCACGGGGGTTGTG
JL23-K114R-F	GAAGGGGAGGTACAACACCACCCTGCTGAACG
JL23-K114R-R	TGTTGTACCTCCCCTTCACCACGGGGGTTGTG
JL23-K114M-F	GAAGGGGATGTACAACACCACCCTGCTGAACG
JL23-K114M-R	TGTTGTACATCCCCTTCACCACGGGGGTTGTG
JL23-K114N-F	GAAGGGGAACTACAACACCACCCTGCTGAACG
JL23-K114N-R	TGTTGTAGTTCCCCTTCACCACGGGGGTTGTG
H138-N114K-F	GTGAAGGGCAAGTTCAATACAACACTGATTAATC
H138-N114K-R	TGTTGAACTTGCCCTTCACCACAGGTTTGCTG
