# Supplementary material for: Preparation and epitope mapping of broad-spectrum neutralizing monoclonal antibodies against economically important pestiviruses
Source: Vet Res. 2026 May 15;57:74. doi: 10.1186/s13567-026-01748-4 (PMC13179623; doi:10.1186/s13567-026-01748-4)
Supplement: Supplementary file 3 — Additional file 3. Primers used for the construction and validation of the recombinant CSFV strains. [file 13567_2026_1748_MOESM3_ESM.pdf]

Additional file 3. Primers used for design and validate the recombinant CSFV strains

Primer	Sequence (5’-3’)
JL23-F1-F	GTATACGAGATTAGCTCATCCTCGT
JL23-F1-R	TCTTTTTTACTTGGTATTTTACTCCCT
JL23-F2-F	GGAGTAAAATACCAAGTAAAAAAGAA
JL23-F2-R	GCCACCTTAGTTTCATACGGCCA
JL23-F3-F	GGTGGCCGTATGAAACTAAGGTGGC
JL23-F3-R	GCACCCAACCAAGGTGCTCCAAATC
JL23-F4-F	GATTTGGAGCACCTTGGTTGG
JL23-F4-R	ACATAGTCAGCTACAGTATTCATTGT
JL23-F5-F	CAATGAATACTGTAGCTGACTATGTGA
JL23-F5-R	ATAGAATATACTTTAGCGCCAACCT
JL23-F6-F	AGGTTGGCGCTAAAGTATATTCTATG
JL23-F6-R	GGGCCGTTAGGAAATTGCCTTA
JL23-K114M-F	GGGATGTACAATACCACTCTATTAAACGGTAGTGC
JL23-K114M-R	GTGGTATTGTACATCCCTTTTACCACTGGGGTTGT
JL23-K114N-F	GGGAACTACAATACCACTCTATTAAACGGTAGTGC
JL23-K114N-R	GTGGTATTGTAGTTCCCTTTTACCACTGGGGTTGT
JL23-K114R-F	GGGAGATACAATACCACTCTATTAAACGGTAGTGC
JL23-K114R-R	GTGGTATTGTATCTCCCTTTTACCACTGGGGTTGT
JL23-K114T-F	AAGGGACGTACAATACCACTCTATTAAACGGTAGTGC
JL23-K114T-R	GGTATTGTACGTCCCTTTTACCACTGGGGTTGT
CSFV-E2-WF	CAGCTSAAYCTAACAGTRGRAC
CSFV-E2-WR	CRCTRAYCATBAGCAAYGCYACYG
CSFV-E2-NF	GGYRAATATGTGTGTGTWAGACC
CSFV-E2-NR	TGGTCTTRACTGGRTTGTTRGTC
