# Supplementary material for: Preparation and epitope mapping of broad-spectrum neutralizing monoclonal antibodies against economically important pestiviruses
Source: Vet Res. 2026 May 15;57:74. doi: 10.1186/s13567-026-01748-4 (PMC13179623; doi:10.1186/s13567-026-01748-4)
Supplement: Supplementary file 4 — Additional file 4. Reactivity of the site-mutant E2 proteins with mAbs TCH034 and TCH052. The CSFV-JL23 E2 protein, which naturally reacts with mAbs TCH034 and TCH052, was subjected to SDM. Specifically, residue G113 in the JL23 strain E2 protein was mutated to V113, while residue K114 was mutated to T114, R114, M114, and N114, respectively. The E2 proteins with amino acid substitutions at 113 and 114 were transiently expressed in HEK293T cells as well as the E2 protein (N114K) of GPeV strain H138, and 20 μg of total protein in the culture supernatants for each protein was subjected to SDS-PAGE and western blot. As a result, the epitope-localized mutations abrogated the binding of the JL23 E2 proteins to both mAbs. In parallel, the N114K mutation in the E2 protein of GPeV strain H138 conferring binding reactivity to both mAbs. [file 13567_2026_1748_MOESM4_ESM.pdf]

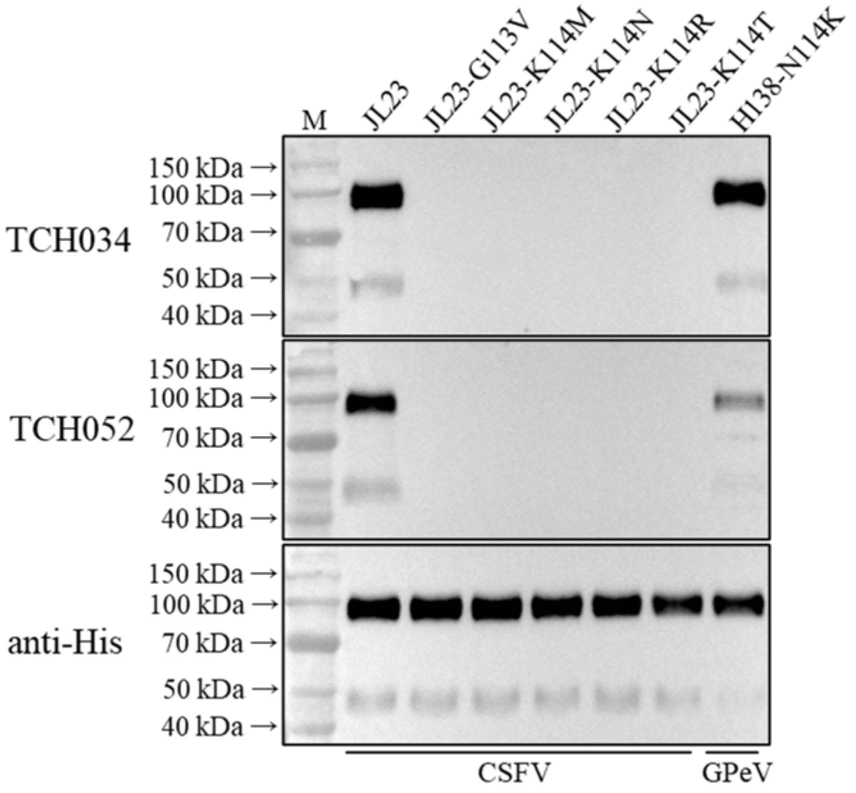

Reactivity of the site-mutant E2 proteins with mAbs TCH034 and TCH052. The CSFV-JL23 E2 protein, which naturally reacts with mAbs TCH034 and TCH052, was subjected to site-directed mutagenesis. Specifically, residue G113 in the JL23 strain E2 protein was mutated to V^{113}, while residue K^{114} was mutated to T^{114}, R^{114}, M^{114}, and N^{114}, respectively. The E2 proteins with aa substitutions at 113 and 114 were transiently expressed in HEK293T cells as well as the E2 protein (N114K) of GPeV strain H318, and 20 μg of total protein in the culture supernatants for each protein was subjected to SDS-PAGE and Western blot. As a result, the epitope-localized mutations abrogated the binding of the JL23 E2 proteins to both mAbs. In parallel, the N114K mutation in the E2 protein of GPeV strain H318 conferring binding reactivity to both mAbs.
